# X-Linked Hypohidrotic Ectodermal Dysplasia in Crossbred Beef Cattle Due to a Large Deletion in *EDA*

**DOI:** 10.3390/ani11030657

**Published:** 2021-03-02

**Authors:** Donal O’Toole, Irene M. Häfliger, Fabienne Leuthard, Brant Schumaker, Lynn Steadman, Brian Murphy, Cord Drögemüller, Tosso Leeb

**Affiliations:** 1 Wyoming State Veterinary Laboratory, University of Wyoming, Laramie, WY 82070, USA; bschumak@uwyo.edu; 2 Institute of Genetics, Vetsuisse Faculty, University of Bern, 3001 Bern, Switzerland; irene.haefliger@vetsuisse.unibe.ch (I.M.H.); fabileuthard@gmail.com (F.L.); cord.droegemueller@vetsuisse.unibe.ch (C.D.); tosso.leeb@vetsuisse.unibe.ch (T.L.); 3 Dermfocus, University of Bern, 3001 Bern, Switzerland; 4 Chadron Veterinary Clinic, Chadron, NE 69337, USA; lynns@panhandle.net; 5 Department of Pathology, Microbiology and Immunology, School of Veterinary Medicine, University of California, Davis, CA 95616, USA; bmurphy@ucdavis.edu

**Keywords:** *Bos taurus*, cattle, ectodysplasin, hypodontia, hypotrichosis, oligodontia, whole genome sequencing, development, dermatology, genodermatosis

## Abstract

**Simple Summary:**

Ectodermal dysplasias such as hypohidrotic ectodermal dysplasia (HED), are genetic conditions affecting the development and/or homeostasis of two or more ectodermal derivatives, including hair, teeth, nails, and eccrine glands. In particular, X-linked hypohidrotic ectodermal dysplasia-1 (ECTD1) in humans is characterized by a triad of signs comprising sparse hair, abnormal teeth, and anhidrosis or hypohidrosis. It has been reported in cattle, dogs, mice and rats. Until now, eight pathogenic variants in the bovine ectodysplasin A (*EDA*) gene causing ECTD1-like disorders have been found. Herein, five affected Red Angus-Simmental bull calves born over a 6-year period (2013–2019) in a single herd in the Western United States are reported showing an ECTD1-like syndrome. Calves were born with severe hypotrichosis and oligodontia. At age 1-week-old two calves died of severe pneumonia. Microscopic findings of the skin revealed small-caliber hair follicles with a mean density in flank skin slightly greater in affected animals than in control animals. Nasolabial, intranasal and tracheobronchial mucosal glands were absent, whereas olfactory glands were unaffected. Whole-genome sequencing (WGS) identified a 53 kb deletion of the X chromosome including parts of the *EDA* gene as well as the entire *AWAT2* gene. The partial deletion of the *EDA* gene that is known to be associated with forms of ECTD1 is the most likely cause for the reported genodermatosis. Similar rare disorders in livestock are often not diagnosed at the molecular level due to lack of resources, short lifespan of the animals, and concerns for the producers’ reputation.

**Abstract:**

X-linked hypohidrotic ectodermal dysplasia-1 (ECTD1) in people results in a spectrum of abnormalities, most importantly hypotrichosis, anodontia/oligodontia, and absent or defective ectodermally derived glands. Five Red Angus-Simmental calves born over a 6-year period demonstrated severe hypotrichosis and were diagnosed as affected with ECTD1-like syndrome. Two died of severe pneumonia within a week of birth. The skin of three affected calves revealed a predominance of histologically unremarkable small-caliber hair follicles. Larger follicles (>50 µm) containing medullated hairs (including guard and tactile hairs) were largely restricted to the muzzle, chin, tail, eyelids, tragus and distal portions of the limbs and tail. The mean histological density of hair follicles in flank skin of two affected calves was slightly greater than that in two unaffected calves. One affected calf was examined postmortem at 10 days of age to better characterize systemic lesions. Nasolabial, intranasal and tracheobronchial mucosal glands were absent, whereas olfactory glands were unaffected. Mandibular incisor teeth were absent. Premolar teeth were unerupted and widely spaced. Other than oligodontia, histological changes in teeth were modest, featuring multifocal disorganization of ameloblasts, new bone formation in dental alveoli, and small aggregates of osteodentin and cementum at the margins of the enamel organ. A 52,780 base pair deletion spanning six out of eight coding exons of *EDA* and all of *AWAT2* was identified. Partial deletion of the *EDA* gene is the presumed basis for the reported X-chromosomal recessive inherited genodermatosis.

## 1. Introduction

Ectodermal dysplasias are inherited diseases in which development and/or homeostasis of two or more ectodermal derivatives such as hair, teeth, sweat and other glands, and occasionally nails, are abnormal [1]. The most common ectodermal dysplasia in people is hypohidrotic ectodermal dysplasia (HED) [2]. Initially classified by clinical presentation and mode of inheritance, members of the HED group are categorized by genotype and molecular pathway, in addition to phenotype [2]. X-linked hypohidrotic ectodermal dysplasia-1 (ECTD1) (OMIM 305100) is caused by variants in *EDA*, which encodes the transmembrane protein ectodysplasin A (EDA), a member of the TNFα-related signaling pathway [3]. Charles Darwin was among the first to describe what later proved to be ECTD1 in a kindred of toothless men from Sind, noting clinically relevant features such as oligodontia, hairlessness and heat intolerance [4]. More than 200 pathogenic variants in human *EDA* are recognized. Affected individuals suffer from heat intolerance due to a limited ability to sweat. Respiratory infections are common in some human forms of ECTD1 and are attributed to absent and/or reduced numbers of glands in the respiratory tract [5]. Additional clinical problems are dental disease, eczema and xerostomia. Similar clinical forms of HED also occur. These are due to pathogenec variants in the *EDAR* gene encoding the EDA receptor, variants in the *EDARADD* gene encoding the EDAR-associated death domain, and. variants in the *WNT10A* gene encoding the secreted signaling molecule Wnt family member 10A. As a result, there are different autosomal recessive and dominant forms of HED [1]. Various forms of HED can be clinically and pathologically indistinguishable because one signal transduction pathway is involved [3].

HED has been documented in mice, dogs, cattle and rats [6,7,8,9,10,11,12]. Our understanding of EDA-related anhidrotic ectodermal dysplasia is based in large part on the *Tabby* (*Eda^T^*^a^) mouse [11,13,14,15]. The ability to ameliorate or abolish congenital defects of HED using short-term treatment with recombinant ectodysplasin was first demonstrated in *Tabby* mice and refined in a canine model, leading to successful treatment of human fetuses [16,17,18,19]. To date, there are eight reported pathogenic variants of the bovine *EDA* gene that, result in an ECTD1-like disease (OMIA 000543-9913) [8,9,10,20,21,22,23,24,25,26,27]. They are potentially lethal since affected cattle are ill-thrifty due to oligodontia and the heightened risk for pneumonia and hypothermia [20,21,27]. Affected cattle can be kept alive when fed chopped feed and in circumstances where there is limited exposure to extreme weather conditions [22,24,27]. Additionally, an HED syndrome affecting Charolais cattle due to a genetic variant in the *EDAR* gene was reported. Similar to its human counterpart, the bovine *EDAR*-related condition is transmitted as an autosomal recessive trait (OMIA 002128-9913) [28]. There are accounts in the peer-reviewed literature of other HED-like syndromes which are based on clinical signs and lesions alone [29,30,31,32].

Published reports of HED in domestic animals focus on the causative genetic variant so that information about lesions other than hypotrichosis and oligodontia is limited. Studies of affected people, mice and dogs document a pattern of lesions in addition to those in skin and teeth, particularly in glands of the oropharynx, ear and orbit, which contribute to clinical and subclinical disease [13,33,34,35]. Information about histological changes in teeth and respiratory tract of ECTD1-affected cattle is limited [24]. Gross changes in the teeth of affected people and dogs include characteristic conical crowns, hypodontia and/or oligodontia, malocclusion, delayed eruption, persisting deciduous teeth, missing permanent teeth, decreased number of cusps, and decreased or diminutive tooth roots [36,37,38,39]. Comparable dental anomalies affect *Tabby* mice [14]

The aim of this study was to characterize the major systemic lesions in a small group of affected Red Angus-Simmental cattle born over a 6-year period in a single herd, and to identify the causative genetic variant.

## 2. Materials and Methods

### 2.1. Herd history and Clinical Investigation

Five crossbred bull calves were born with hypotrichosis and oligodontia over a 6-year period (2013–2019) in a commercial Red Angus-Simmental herd in western Nebraska, USA. Both natural breeding and artificial insemination were used. Each calf was born to a unique dam and was full-term based on the owner’s assessment of body size and birth weight. The owner changed ear tags once he decided to retain animals in the herd. No key was kept for the two sets of identification numbers so it was not possible to generate a pedigree of the dams. Each of the five dams was clinically normal, according to the owner, although no examination of the oral cavity was performed to check for abnormal dentition in four of the five cattle. Skin biopsies were collected from three affected calves (cases 1 and 2 in 2013; case 3 in 2019; cases 4 and 5 were assessed by clinical examination alone) by a veterinary practitioner (L.S.) in order to establish a diagnosis for alopecia. After case 3 was born in 2019, the owner examined the incisors of this fifth dam, a first-calf heifer, and found no abnormalities such as missing or malformed teeth. The dam’s cheek teeth (premolars and molars) were not examined. Case 3 was kept indoors from birth in a heated barn, and wrapped in an insulated coat to prevent pneumonia. Once it was established from a skin biopsy that the calf was free of bovine viral diarrhea virus (a recognized cause of congenital alopecia due to in utero infection) and that histological lesions in skin were indistinguishable to those in cases 1 and 2, the calf was humanely euthanized for diagnostic purposes at 10 days after birth. No affected calves were born subsequently into the herd in 2020. As of this writing, a single affected male calf was born into the herd in Feb 2021.

### 2.2. Pathological Investigation

Skin samples (5 × 5 cm) obtained by biopsy from the three affected calves were fixed in 10% buffered formalin. Skin was sampled from one pinna and the dorsal aspect of the neck (case 1), pinna alone (case 2), and pinna, muzzle, flank, upper and lower eyelids, lateral aspect of thigh, distal aspect of forelimbs and hindlimbs, tailhead, and distal aspect of the tail (case 3). Skin samples from calf 3 were selected to include lightly haired areas (e.g., flank) and better haired areas (e.g., distal aspect of limbs and tail; eyelids). A comprehensive set of tissues was collected into 10% formalin from case 3 at necropsy. These included tissues from oral and nasal cavities (tongue; cheek with oral mucosa; hard palate; nasal and ethmoidal conchae), palatine tonsils, larynx, trachea, bronchi, and lung including bronchioles. No samples were collected to evaluate lacrimal, ceruminous or major salivary glands. Maxillae and mandibles of case 3 were radiographed after death, fixed for 7 days in 10% buffered formalin, cut with a band saw along the long axis of teeth, and examined grossly. Teeth, mandibles and maxillae were decalcified in a formic acid-EDTA solution (CalFor*TM, Cancer Diagnostics Inc., Durham, NC, USA). After fixation, tissues were routinely processed through graded alcohols before embedding in paraffin. Sections were cut 5 µm thick and stained with hematoxylin and eosin (HE). Sections of skin from calves 1 and 3 were cut either perpendicular or parallel to the epidermal surface. The diameter of hair follicles was determined by stereology using flank skin from two affected (cases 1 and 3) and two age- and herd-matched Red Angus-Simmental control calves. The cross-sectional area of follicles areas was determined using a 4-point nucleator tool and converted to diameters (Stereo Investigator Software, MBF Bioscience, Williston, VT, USA). The density of hair follicles was measured using commercial image-capturing software (Olympus cellSens, Tokyo, Japan). Sections of aural skin from cases 1–3 were stained immunohistochemically for bovine viral diarrhea virus (BVDV) antigen using appropriate positive and negative controls performed as described by Cornish et al. [40]. For tissues controls, samples of flank skin were collected postmortem from two female Red Angus-Simmental 5- or 6-day-old calves from the same ranch where affected calves were born. Death was due to neonatal disease, based on field necropsies performed by one author (L.S.). One calf had acute pneumonia and the other necrotizing hemorrhagic enteritis. The skull of a phenotypically normal 8-day-old Galloway bull calf that died of myocardial necrosis and interstitial pneumonia served as a control for the histology of normal bovine teeth and mucosa of nasal and ethmoidal conchae. All three control calves had normal pelage and dentition.

### 2.3. Genetic Investigation

Genomic DNA was isolated from the spleen of case 3 using a commercial kit (MagMax; Applied Biosystems/Thermo Fisher, Waltham, MA, USA). An Illumina Truseq PCR-free fragment library of the DNA from case 3 was prepared, generating ~176 million 2 × 150 bp read-pairs corresponding to 17.6× coverage on a NovaSeq 6000 instrument (illumina, San Diego, CA, USA). The reads were mapped to the ARS-UCD1.2 cattle reference genome (GCF_002263795.1) and subsequently single-nucleotide variants (SNVs) and small indel variants were called. The applied software and steps to process fastq files into binary alignment map (BAM) and genomic variant call format files were in accordance with the 1000 Bull Genomes Project processing guidelines (www.1000bullgenomes.com (accessed on 11 February 2021)). Further preparation of the genomic data had been done according to Häfliger et al., 2020 [41]. The Integrative Genomics Viewer (IGV) software was used for visual inspection and identification of structural variants [42]. The whole-genome sequencing (WGS) data are available at the European Nucleotide Archive under study accession PRJEB18113 and sample accession SAMEA5714973 (https://www.ebi.ac.uk/ena/data/view/ERS3518602 (accessed on 11 February 2021)). In order to find private variants, we compared the genotypes of the affected calf with 496 cattle genomes of various breeds that had been sequenced in the course of other ongoing studies at the Institute of Genetics at the Vetsuisse Faculty, University of Bern, and that are publicly available in the European Nucleotide Archive under study accession PRJEB18113 (http://www.ebi.ac.uk/en (accessed on 11 February 2021)).

## 3. Results

### 3.1. Clinicopathological Phenotype

**Figure 1:**
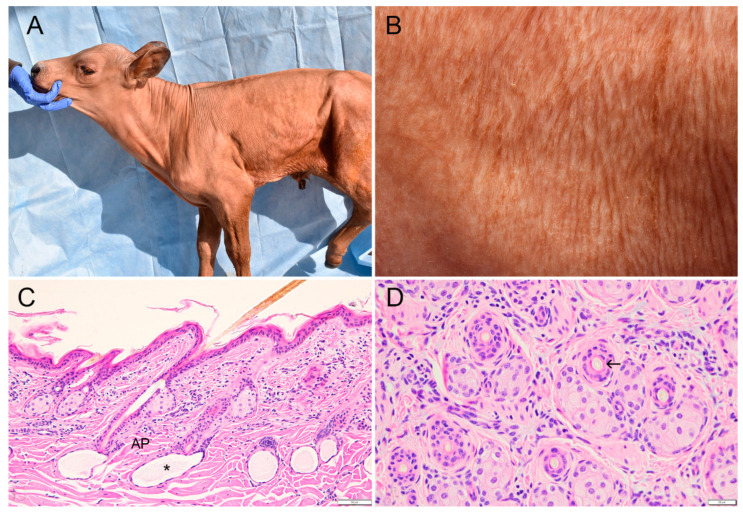
Cutaneous clinicopathological findings in a Red Angus-Simmental calf with hypohidrotic ectodermal dysplasia. (**A**) Case 3 at 10 days of age. Absence of visible hairs on neck, trunk, upper limbs or most of the face, with exception of eyelashes and pinna. (**B**) Case 3 at 10 days of age. Higher magnification of skin from flank showing small fine hairs grouped in linear dorso-ventral patterns. (**C**) Flank skin; case 3. Uniformly small hair follicles with associated sebaceous glands, pigmented hair shafts, and arrector pili muscles (AP). Sweat glands are closely associated with dermal papillae and contain lightly stained secretion (asterisk). Note mild lymphocytic dermatitis in upper to mid-dermis. Bar: 50 µm. (hematoxylin and eosin stain (H&E)). (**D**) Flank skin; case 1. Follicles contain hair shafts (arrow) and are associated with unremarkable sebaceous glands. No orphan sebaceous glands are present. Bar: 25 µm. (H&E).

The five affected calves appeared almost completely hairless at birth, with the exception of eyelashes, muzzle, chin, tragus, and distal parts of the limbs and tail (Figure 1A). Vibrissae were present on the muzzle and chin. Small fine hairs suggestive of undercoat (pili lanei) were evident over the trunk, neck, head and upper two thirds of the limbs (Figure 1B). Digits were unremarkable, including periople and horn.

The histological appearance of hair follicles in the affected calves were indistinguishable from the smallest follicles in the control calves. The majority of hair follicles (>90%) contained lightly pigmented hairs and had a normal relationship with unremarkable arrector pili muscles and sebaceous glands (Figure 1C). Apocrine glands were sac-like with light blue contents in HE-stained sections and were lined by low cuboidal epithelium. They lay deep to hair follicles and were closely associated with the dermal papillae. Depending on the area of skin sampled from calf 3, small hair follicles were either the sole or predominant follicle type present (Figure 1D). This animal, at 10 days the oldest of the three affected calves whose skin was examined histologically, was the only one to have mild diffuse perivascular lymphocytic dermatitis.

**Figure 2:**
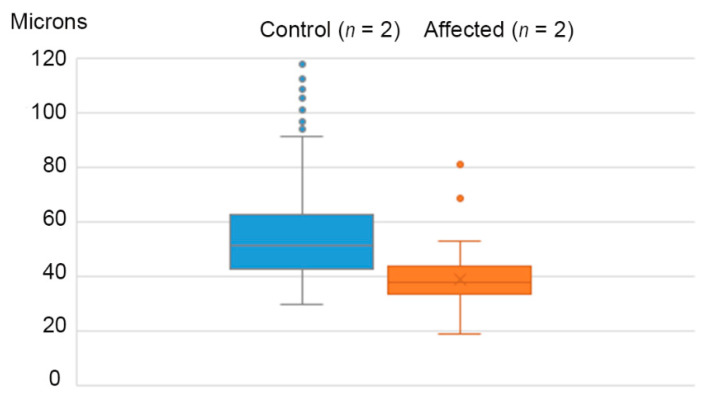
Comparison of median diameter of hair follicles in two control calves (blue) and two affected calves (orange; cases 1 and 3). In the control calves, 14 follicles were >120 µm diameter and not illustrated; they are included in the median. Median follicular diameters are 49.9 and 38.1 µm in unaffected and affected animals, respectively.

The median diameter of hair follicles in flank skin was 36.4 and 38.4 µm in affected calves 1 and 3, compared to 51.1 and 51.3 µm in two control calves (Figure 2). The density of hair follicles in flank skin of the same two affected calves was slightly greater than that of two calves with normal hair coats (Table 1).

Normal Meibomian glands were present in the eyelids; no effort was made to quantify them. Nasolabial glands were absent in the planum of calf 3. The skin of cases 1–3 was negative for BVDV by immunohistochemistry.

**Figure 3:**
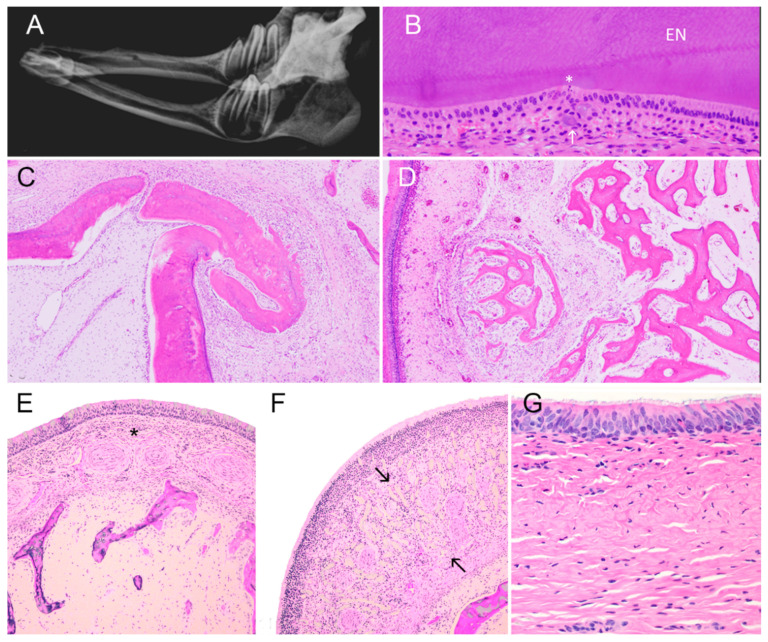
Dental anomalies in a hypohidrotic ectodermal dysplasia-affected calf. (**A**) Mandible (radiograph); case 3. Incisors are absent. Premolar teeth are narrow and lack the complex apical and root structures of cheek teeth in unaffected neonatal calves. No permanent teeth have developed. (**B**) Premolar tooth; case 3. Mildly disordered ameloblasts with scant apoptosis (asterisk) adjacent to enamel (EN). Small amorphous aggregates of mineralized material, probably enamel, in papillary layer (arrow). (H&E stain). (**C**) Premolar tooth; case 3. Disorderly focus of secondary dentin or osteodentin covered with nodular aggregate of cementum projects into alveolus. (H&E stain). (**D**) Premolar tooth; case 3. New bone formation extending into alveolus. (H&E satin). (**E**) Nasal concha, case 3. Mucosal glands, which in normal calves lie between vascular cavernous stratum and the mucosal surface (asterisk), are absent. (H&E stain). (**F**) Ethmoidal concha, case 3. Olfactory glands (between arrows) are present beneath mucosal epithelium. (H&E stain). (**G**) Trachea, case 3. Note the complete absence of tracheal glands. (H&E stain).

Case 3 had 12 deciduous teeth, all of which were unerupted or barely erupted premolars (oligodontia). Normal neonatal calves have 20 deciduous teeth (incisors, canines and premolars) [43]. The dental formula in case 3 was deciduous incisors 0/0, deciduous canines 0/0, deciduous premolars 3/3, and permanent molars 0/0. In normal healthy calves, the crown of the permanent first mandibular molars is generally grossly evident at birth [43]. By contrast, in case 3 by 10 days of age no permanent teeth were evident radiographically, grossly or histologically. Unerupted or barely erupted premolars were slender, slightly curved, and more widely spaced than the closely packed premolars of an 8-day old calf used as a control (Figure 3A). Microscopically, premolar teeth demonstrated all components of normally developing bovine teeth. No dental matrices (dentin/enamel) of incisor, canine or molar teeth were detected histologically. In spite of gross malformation in the shapes of the premolar teeth, histological changes were modest. They comprised focal disorganization of ameloblast palisades, deposition of enamel-like aggregates in stratum intermedium (Figure 3B), disorderly spurs of dentin or osteodentin with cementum (Figure 3C), and limited new bone formation in dental alveoli (Figure 3D). Normal minor salivary glands were present in mucosa of the cheek and palate. Olfactory glands were present in mucosa of the ethmoidal conchae (Figure 3E). By contrast, no tracheal or bronchial-bronchiolar glands were present in the respiratory tract (Figure 3F,G). There was no evidence of inflammation in the upper or lower respiratory tract of case 3.

### 3.2. Genetic Analysis

**Figure 4:**
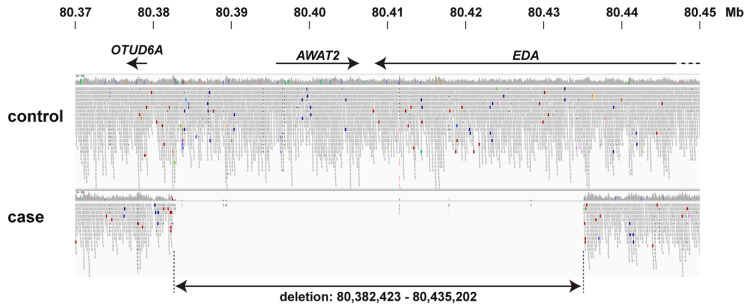
Genetic analysis showing screenshot from Integrative Genome Viewer (IGV) software. The short-read alignments from whole genome sequencing of an affected calf (case 3) and a normal newborn calf are shown. The affected calf lacks read coverage in central region due to a ~53 kb genomic deletion harboring the entire *AWAT2* gene and the last 6 exons of EDA.

Filtering of the obtained variant catalogue for private variants exclusively present in the genome of the affected calf and absent in 496 available control genomes identified any private protein-changing variants. As our automated variant calling was limited to single nucleotide and small indel variants, we additionally performed a visual analysis for large structural variants in four known HED candidate genes (*EDA, EDAR, EDARADD, WNT10A*). This analysis revealed a large ~53 kb deletion on the X chromosome in the affected calf 3. It can be formally designated as ChrX:80,382,423_80,435,202del52,780 (ARS-UCD1.2 assembly). The deletion was flanked by highly similar sequences that showed 95% sequence identity over 259 bp suggesting that the deletion may have arisen during an unequal crossing over event. The deleted region encompassed the entire *AWAT2* gene and the last six exons of the *EDA* gene (Figure 4).

## 4. Discussion

The lesions in skin, teeth and respiratory tract of affected calves closely resembled those reported in cattle with other forms of EDA-related hypohidrotic ectodermal dysplasia. Genetic analysis revealed a large X-chromosomal deletion involving the *EDA* gene.

The most striking gross abnormality in calves was in skin. Guard hairs were absent or scant over much of the trunk, neck, head and proximal part of the limbs and tail. Reports of lesions in other forms of bovine ECTD1 describe underdeveloped or hypoplastic hair follicles, fewer or abnormal sebaceous glands, absent eccrine nasolabial glands in the muzzle, and abnormal or absent apocrine sweat glands [20,22,27]. With the exception of their slightly smaller size, the predominant type of hair follicle was indistinguishable from undercoat follicles in the control calves that had normal hair coats. These may correspond to small “last formed follicles” which develop in late gestation [44]. As with ECTD1 in people and the *Tabby* mouse, the lack of functional ectodysplasin A during a key period in gestation resulted in failure of larger follicles to develop normally in most areas of the skin [17,45]. The density of hair follicles in the affected calves presented in this study was only slightly greater than in unaffected calves. This is dissimilar to a calf with ECTD1, which had five times more hair follicles than a control calf [10]—this might be due to using different skin sites (scalp vs. flank) for follicular counts in the two variants of ECTD1. Sebaceous glands in the herein studied calves were morphologically normal. Glands of the upper and lower respiratory tract were also absent, as reported in other calves with ECTD1 [9].

Apocrine sweat glands were present, generally closely associated with dermal papillae. Eccrine sweat glands are markedly reduced in ECTD1 in people, resulting in susceptibility to hyperthermia [9,35]. In previously reported cases of bovine ECTD1, apocrine glands were reported to be absent, reduced in number, slightly dilated or underdeveloped, or to have flattened epithelium suggestive of inactivity [8,9,10,20,22,24]. The morphology of apocrine glands in affected Red Angus-Simmental calves resembled that of control calves. The shape and size of normal bovine sweat glands is variable, determined in part by breed [46]. Unlike eccrine sweat glands of people [47] and mice [48], there is no simple way to assess the function of apocrine sweat glands in newborn calves [49]. We did not attempt to quantify sweat glands in skin from different areas.

Not surprisingly, similarities exist between the phenotype of ECTD1 in affected cattle and that of dogs, mice and humans. This includes the distribution of hairless and lightly haired areas (cattle; dog) and a predisposition to respiratory infection (cattle; dog; human) [35,50]. The distribution of hypotrichosis differs between calves and dogs in that it is generally more extensive in cattle where it generally affects facial skin, dorsal, lateral and ventral parts the neck and trunk, and the proximal portions of limbs. The distribution of ECTD1 hypotrichosis in dogs tends to be more restricted [12,37,39,51]. Although no comprehensive assessment of glands was attempted in the current study, mucosal glands were absent in upper and lower portions of the respiratory tract. As in the *Tabby* mouse [15], the olfactory glands of at least one affected Nebraska calf had developed normally. Recurrent conjunctivitis is described in some species with ECTD1 and variously ascribed to reduced tear production (dogs) [39] or a primary keratopathy (*Tabby* mouse) [33]. This has not been reported in cattle, possibly because most affected calves die or are killed so soon after birth. No clinical lesions were evident in the eyes of the five Nebraska calves. Ocular lesions were absent in one calf, which died at 10 days of age. Meibomian glands were present in his eyelids.

Deciduous incisor teeth of full-term calves erupt between birth and 21 days post-partum [43]. Incisor and canine teeth were radiographically, grossly and histologically absent in the one affected calf whose teeth were examined in detail. For these teeth, no nascent dental follicles, enamel organs, mineralized dental matrices, or dental ectomesenchyme were identified in the jaws. Premolar deciduous teeth were abnormally slender and more widely spaced than in a normal calf. The examined dental follicles of deciduous teeth did not demonstrate any overarching defect in odontoblasts, ameloblasts, cementoblasts or associated mesenchyme. This is consistent with earlier findings in Holstein calves with ECTD1 and in the *Tabby* mouse, where the principal histological dental abnormalities were oligodontia, delayed eruption, and defective dental shape, size and orientation [14,22,24]. Early in odontogenesis, however, delayed dental differentiation in *Tabby* was associated with degeneration of ameloblasts, as well as altered apoptosis patterns [52,53].

It is recognized that, in addition to abnormal development of skin and teeth, humans with ECTD1 lack normal glands in the respiratory tract, predisposing patients to nasal obstruction, sinusitis, and respiratory infections [34]. Similar changes affect the *Tabby* mouse [7] and cattle with ECTD1 [10,20,27]. Interestingly, pneumonia was responsible for the death of two of five affected calves on the affected ranch. Of the remaining three affected calves, two died without being examined postmortem to determine a cause of death. The remaining calf (case 3) was kept alive indoors until euthanasia.

The genetic investigation revealed a plausible candidate causative variant located on the X chromosome since a large part of the *EDA* gene was deleted in the sequenced affected calf. Interestingly, the *EDA* gene along with the neighboring *AWAT2* gene encoding acyl-CoA wax alcohol acyltransferase 2 was affected. *AWAT2* catalyzes the formation of wax monoesters in sebocytes [54]. The presumptive inactivation of *AWAT2* did not result in any detectable lesion in sebaceous glands. A genetic form of ECTD1 with a deletion that encompassed *EDA* and *AWAT2* has been reported in a human patient. Similar to the calves reported here, no additional clinical features were evident in that individual [55].

## 5. Conclusions

This represents the first form of X-linked hypohidrotic ectodermal dysplasia in cattle that affected both *EDA* and *AWAT2* genes. Regardless of the *EDA* variation, affected calves are clinically and morphologically similar, and possess many of the lesions found in affected human patients. For many genes it is known that the kind of genetic alteration influences the phenotypic outcome, e.g., the severity of a congenital defect varies or differs totally, depending on the individual variant. Interestingly for *EDA* in cattle this seems not to be the case as different kinds of variants always cause an essentially identical phenotype which is of importance for diagnostic pathologists. Furthermore, rare disorders, such as ECTD1-like syndrome in livestock are mostly not diagnosed at the molecular level, due to a lack of resources, the often-short lifespan of the animals, and concerns that producers have for their reputation as conscientious breeders. This example highlights the utility of WGS-based precise diagnostics for understanding rare disorders in animals with an available reference genome sequence, and the need for continued surveillance for genetic disorders in cattle breeding. Genome sequencing might improve the precision of the clinicopathological diagnosis as sometimes unexpected variants in genes that were not known to be associated with a certain disorder can be detected.

## Figures and Tables

**Table 1:** Density of hair follicles in flank skin of affected and unaffected calves.

Calves		Number of Hair Follicles/Area (153,137 µm^2^) ^a^			Mean Follicle Density/Area (153,137 µm^2^)	Mean Density Follicles/mm^2^
		Area 1	Area 2	Area 3		
Affected	No. 1	30	31	30	30.3	164.50
Affected ^b^	No. 3	22	18	23	21.0	
Unaffected ^c^	No. 1	20	23	22	21.7	133.9
Unaffected ^d^	No. 2	18	21	19	19.3	

^a^: Measured in skin from flank in all 4 calves. ^b^: Mild diffuse lymphocytic dermatitis in this calf may have slightly reduced follicle density. ^c^: Died at 5 days of age of pneumonia; normal hair coat. ^d^: Died at 6 days of age of necrotizing enteritis; normal hair coat.

## Data Availability

The whole-genome data of the affected calf is freely available at the European Nucleotide Archive (ENA) under sample accession number SAMEA5714973.
